# Context and target recollection for words and pictures in young adults with developmental dyslexia

**DOI:** 10.3389/fpsyg.2022.993384

**Published:** 2022-12-05

**Authors:** Michał Obidziński, Marek Nieznański

**Affiliations:** Institute of Psychology, Faculty of Christian Philosophy, Cardinal Stefan Wyszyński University, Warsaw, Poland

**Keywords:** developmental dyslexia, target memory, context memory, dual recollection theory, multinomial modeling

## Abstract

**Introduction:**

The specificity of memory functioning in developmental dyslexia is well known and intensively studied. However, most research has been devoted to working memory, and many uncertain issues about episodic memory remain practically unexplored. Moreover, most studies have investigated memory in children and adolescents—much less research has been conducted on adults. The presented study explored the specificity of context and target memory functioning for verbal and nonverbal stimuli in young adults with developmental dyslexia.

**Methods:**

The dual recollection theory, which distinguishes context recollection, target recollection, and familiarity as the processes underlying memory performance in the conjoint recognition paradigm, was adopted as the theoretical basis for the analysis of memory processes. The employed measurement model, a multinomial processing tree model, allowed us to assess the individual contributions of the basic memory processes to memory task performance.

**Results:**

The research sample consisted of 82 young adults (41 with diagnosed dyslexia). The results showed significant differences in both verbal and nonverbal memory and context and target recollection between the dyslexic and the typically developing groups. These differences are not global; they only involve specific memory processes.

**Discussion:**

In line with previous studies using multinomial modeling, this shows that memory functioning in dyslexia cannot be characterized as a simple impairment but is a much more complex phenomenon that includes compensatory mechanisms. Implications of the findings and possible limitations are discussed, pointing to the need for further investigation of the relationship between context memory functioning and developmental dyslexia, taking into account the type of material being processed.

## Introduction

Developmental dyslexia (DD) is a type of neurodevelopmental disorder, a specific learning disorder/developmental learning disorder (*cf*. American Psychiatric Association, 2013; World Health Organization, 2022), characterized by impaired fluency and accuracy in the reading process and difficulties with spelling, but not by problems with reading comprehension. As with many other disorders, despite the number of studies and years of searching for treatment methods, our knowledge about DD, as well as its etiology and pathomechanisms, is still lacking. This fact encourages further studies and theoretical explorations to establish adequate and useful definitions and models for the discussed disorder. The problems experienced by people with dyslexia do not seem to be limited to school education and persist into adulthood (e.g., Jones et al., 2009; Łockiewicz et al., 2012; Warmington et al., 2013; Bogdanowicz et al., 2014; Reis et al., 2020). Moreover, some studies suggest that there is a change between childhood and adulthood in which behavioral problems become central difficulties (*cf*. Everatt, 1997; Łockiewicz and Bogdanowicz, 2013). This observation has motivated even more research into developmental dyslexia, focusing on the experiences of adults with diagnosed DD.

### Memory in dyslexia: An overview

Over the years of research on developmental dyslexia, numerous theories and hypotheses about the mechanisms of the disorder have been proposed and tested, such as the visual hypothesis (Eden et al., 1996), the verbal hypothesis (Vellutino, 1977), the phonological hypothesis (Snowling, 1998), the magnocellular hypothesis (Livingstone et al., 1991), or the dual processing hypothesis (Bowers and Wolf, 1993), to name a few. Many publications present the historical and current status of these endeavors, where readers can find a more or less detailed description of all the major concepts (e.g., Stein, 2018; Snowling, 2019; Kuerten et al., 2020). In this paper, a broader description will be devoted to the ideas about memory functioning as a pathomechanism and/or compensatory mechanism for developmental dyslexia.

Memory functioning impairment is one of the best-documented differences between individuals with dyslexia and their typically developing (TD) peers (Snowling, 2019). Since the beginning of research on specific learning disorders, hypotheses about memory impairment have been put forward and investigated and there is an overwhelming amount of evidence for verbal memory impairments in developmental dyslexia. Numerous studies have shown this using the digit span task or other verbal short-term or working memory tasks (e.g., Varvara et al., 2014). An impairment of memory for verbal material can be found not only in short-term memory functioning but also in long-term memory performance (e.g., Menghini et al., 2010; Obidziński and Nieznański, 2017). The results regarding the functioning of nonverbal memory—mainly visual and spatial—are mixed. In some of the studies, authors report significant impairments of memory for other stimuli modalities, namely visual and visuo-spatial (e.g., Smith-Spark et al., 2003; Smith-Spark and Fisk, 2007; Bacon et al., 2013). However, there is also a number of studies showing differences only for verbal memory, with a normal performance in other modalities, or suggesting that there may be a visual subtype of dyslexia, but that these impairments are not a common trait of all individuals with DD (e.g., Del Giudice et al., 2000; Jeffries and Everatt, 2004; Giofrè et al., 2019). Interestingly, some authors report an enhancement of visuo-spatial abilities in dyslexia and propose the hypothesis of visuo-spatial superiority in DD (e.g., Brunswick et al., 2010; Bacon and Handley, 2014). Therefore, the study of non-verbal memory performance in dyslexia should not only focus on possible impairments but also on strengths—which can be seen as a form of compensatory mechanism. Based on the reviewed literature, it can be concluded that there are significant differences in the long-term memory performance of DD and TD groups. Impairments of episodic memory were found not only for verbal material but also for visual and spatial material (e.g., Menghini et al., 2010). Moreover, specific false memory effects (FM) were found (see: Obidziński and Nieznański, 2017; Obidziński, 2021) showing that the DD group is more prone to FM when presented with orthographically related distractors. Overall, on the one hand, studies of memory in DD clearly show differences in its functioning (compared to TD), but on the other hand, many conflicting results lead to competing hypotheses about the exact extent of memory differences.

As can be seen from the above, most studies exploring the presented issue focus on working memory and are based on classical theoretical frameworks. Therefore, despite numerous papers on memory functioning in dyslexia, new studies could explore this relationship more deeply and broadly using theoretical frameworks that are better suited to the specifics of this developmental disorder. Moreover, most studies use standard statistical measures rather than modeling or data mining analyses. This limits access to data that could be important for a better understanding of the specificity of dyslexic memory processes. New studies are needed that will use contemporary analytical methodology to better understand the issue under study. A good example of this approach—both at the level of theory and methodology—are studies using fuzzy-trace theory (FTT) to investigate the memory functioning differences in developmental dyslexia (e.g., Obidziński and Nieznański, 2017; Obidziński, 2021).

### Verbatim and gist memory in dyslexia

Fuzzy-trace theory (e.g., Brainerd and Reyna, 1990, 2002, 2015) is a theory of memory and decision-making that assumes that there are two parallel but independent memory traces for each stimulus—in contrast to the classical approach, which assumes a unitary memory trace. The first, verbatim trace, involves encoding surface (mainly perceptual) information about the perceived stimulus (such as colors, shapes, sounds, etc.). The second, the gist trace, involves encoding deeper information (such as the meaning or semantic relationship). For example, if we see the word “doctor” written on a piece of paper, the verbatim trace will encode information about the phonological and visual characteristics of the word, and the gist trace will encode that “it is a word naming a person with a medical degree who helps ill people, its synonym is physician, etc.” FTT plays an important role in false memory research (e.g., Brainerd and Reyna, 2002, 2019; Reyna et al., 2016), which proposes that it is not only mechanisms of memory distortion but also memory processes that counteract FM (phantom recollection and recollection rejection, respectively; Brainerd et al., 2001, 2003). A process that leads to FM based on strong gist trace retrieval is called *phantom recollection* (Brainerd et al., 2001). It is a memory illusion that a distractor was certainly presented in the study phase. On the other hand, a process called *recollection rejection* is based on verbatim trace retrieval, and it counteracts FM. During the test phase, the presentation of a related distractor may lead to the retrieval of a verbatim trace of a corresponding target, which results in correct rejection of the distractor due to noticing that it is similar but not identical to the target. FTT also describes developmental trajectories of memory functioning—which provides a better understanding of the FM and decision-making effects observed in human development (*cf*. Reyna, 2012).

The multinomial model for the conjoint recognition paradigm, which is the main method used in FTT research, was created in several different variants, starting with the first model proposed by Brainerd et al. (1999), through its simplified version proposed by Stahl and Klauer (2008, 2009), and further developments prepared by Brainerd et al. (2010) and Brainerd et al. (2021). In all of these versions, three types of test stimuli are used: old items (targets), unrelated distractors, and related distractors. In a simplified model (Stahl and Klauer, 2008, 2009), participants are asked in the test phase of the experiment to decide if the presented stimuli are old, new but related to old, or entirely new (a forced-choice between three options). The triad procedure modification of the simplified model adds perceptually similar test stimuli to the procedure (Obidziński and Nieznański, 2017). This way, with two separate study lists and test phases, the modified procedure allows the observation of gist and verbatim memory functioning concerning both semantic and perceptual similarity. For this reason, the resulting model consists of a doubled number of multinomial trees and parameters in comparison with the simplified model (Stahl and Klauer, 2009), half of which relate to the processing of perceptual stimuli.

Because of the distinction between verbatim and gist traces, FTT can be intuitively connected with difficulties observed in developmental dyslexia (*cf*. Miles et al., 2006; Obidziński and Nieznański, 2017). It should be noted, however, that the proposed link goes beyond the standard account of memory systems. Typically, word perception/recognition during the reading process is referred to as the specific memory system (i.e., the perceptual representation system and the visual word form subsystem: see Schacter, 1994) which is separate from the episodic memory system. However, in this paper we want to go beyond the distinctions between memory systems and take the general perspective of FTT (we also proposed this in our previous paper: Obidziński and Nieznański, 2017). Verbatim traces processing seems to be important for fast and accurate reading because remembering the relationship between the graphical and phonological traits of written language is necessary for efficient reading. Therefore, an impaired encoding of verbatim traces can lead to problems in orthographic details processing that are important for the accurate reading of text but not for its comprehension. Furthermore, the pattern of memory process contribution to performance, measured using multinomial modeling, shows that an impairment is observed in only one of the two verbatim processes, namely recollection rejection, and, thus, in the process that is involved in counteracting FM (Obidziński and Nieznański, 2017). This fact is consistent with the problems observed in DD, namely the inaccurate reading of words. When a written word is seen and the reader’s mind searches for memorized syllables or words to pronounce the word, there is a possibility that the memory will find words that are pronounced very similarly but not in the same way. Therefore, it is necessary to employ certain control mechanisms that will help to differentiate between representations of similar items and target traces—this process is recollection rejection. In this proposed model of interaction between memory and reading, memory impairment will lead to less accurate reading because similar pronunciations will be accepted as correct ones.

Therefore, an analysis of more elementary memory processes—as described in the conjoint recognition model and its modifications (e.g., Brainerd et al., 1999; Stahl and Klauer, 2009; Obidziński and Nieznański, 2017)—shows that the FTT theoretical framework captures not only the specificity of DD symptoms but also fits the reading process (Obidziński and Nieznański, 2017). Despite this fact, only a few studies on memory in DD have incorporated the FTT framework, and even fewer have used the modeling approach (Miles et al., 2006; Chechile, 2007; Blau, 2013; Voss, 2013; Obidziński and Nieznański, 2017). Nevertheless, the conducted studies have provided data that not only offer new insights into the potential role of memory in dyslexia symptoms but also (in the case of the modeling approach) allow the inconsistent results reported in studies using standard analyses to be explained (Obidziński and Nieznański, 2017).

### Context and item recollection in dyslexia

Dual recollection theory (DRT) is a theoretical approach developed from FTT that separates the processes of target and context recollection (Brainerd et al., 2014, 2015). Human memory encodes not only core information about the stimulus itself (e.g., a word or picture), but also the context of its presentation (e.g., the font color and the sounds in a study room). Context information is used in different ways, for example, as a probe for other information (e.g., Chun and Jiang, 1998; Smith et al., 2014) or in reality monitoring processes (e.g., Kensinger and Schacter, 2006). According to most dual-memory models (Yonelinas, 2002; Malmberg, 2008), recollection reflects the conscious reinstatement of details from a learning episode, including both target and contextual information. In contrast, the DRT proposes that the processes of target recollection and context recollection are two separate and parallel processes. Thus, the DRT framework can predict the specific effects of memory, like recollecting the context without recollecting the target (e.g., Chen et al., 2018), which are unexplainable in theoretical approaches that assume the existence of only one recollection process.

In the multinomial model for DRT proposed by Brainerd et al. (2015), the following retrieval processes are defined by the model parameters: (a) target recollection *T*, which is the probability that a target cue provokes the conscious reinstatement of its presentation during study and acceptance of the proffered source; (b) context recollection *C*, which is the probability that a target cue from a specific source provokes the conscious reinstatement of the contextual details of this particular source’s presentation; (c) familiarity *F*, which represents the probability that a target cue provokes a sufficiently high familiarity to make the target be perceived as old; and (d) response biases *b* and *bPS*, which are the probabilities of accepting a nonpresented cue (or unrecognized target cue), depending on the kind of probe question. Details of this model, as applied to the current study, will be presented in the section “Materials and methods.”

It must be noted that DRT is directly connected with FTT, however, these two theories are not interchangeable. As recently noted by Brainerd et al. (2021), FTT introduces two memory traces (verbatim and gist) instead of one memory trace, while DRT assumes yet another, third kind of trace—a context trace. Therefore, processes assumed in FTT become a basis for some of the processes assumed in DRT, but not all of them. As a result, some processes postulated by DRT can be treated as equivalents to those of FTT. Thus, the effects observed in the FTT study for these specific processes should also be observed in the parameters of the DRT model. Among processes that can be treated as equivalent are: recollection rejection and verbatim trace retrieval which correspond to target recollection, phantom recollection which corresponds to context recollection, and gist trace retrieval which corresponds to semantic familiarity.

The bivariate concept of recollection allows for the investigation of how different variables affect these separate processes and, therefore, how effective the retrieval of different types of realistic information is under specific conditions. As previous studies have suggested, using the DRT approach to the study of memory functioning in the general population can highlight differences depending on, for example, verbal vs. non-verbal stimuli, the level of processing (Nieznański, 2020) or other variables (e.g., Niedziałkowska and Nieznański, 2021). The use of the DRT model allows the parameters to be estimated for both recollection types using either the multinomial processing tree model or the signal detection model. Developing FTT into the dual recollection theory may further extend our knowledge about the specificity of memory functioning in DD. Taking into account a recent meta-analysis and theoretical advances in dual-recollection theory, we will follow the terminology and concepts proposed by Brainerd et al. (2021).

Despite the lack of studies investigating context memory/source monitoring in DD, there are findings and theoretical concepts that can be used to argue the relevance of context memory for the issue being presented. First, the severity of the problems with reading and writing words is much lower than the problems observed for pseudowords in older children, adolescents, and adults with dyslexia (e.g., Snowling et al., 1994; Taroyan and Nicolson, 2009). Therefore, a word having formal features but lacking semantic and contextual information seems to be much more problematic in verbal processing for the dyslexic group. Furthermore, although both typically developing and dyslexic individuals use contextual information to monitor text during the reading process and facilitate words that will be used in order to enhance fluency and accuracy, contextual facilitation is harnessed more often by people with DD (e.g., Nation and Snowling, 1998; Vellutino et al., 2004).

As mentioned earlier, a pattern of strengthening gist memory was observed in studies using FTT (e.g., Miles et al., 2006; Obidziński and Nieznański, 2017). Initially, context recollection was defined as based on gist trace retrieval (e.g., Brainerd et al., 2015). However, a recent meta-analysis conducted on a large set of conjoint recognition studies (Brainerd et al., 2021) showed that the three-factor model fits the data better, where the context information is stored in a separate context trace. Therefore, on the one hand, the findings of the differences in memory functioning in dyslexia for gist traces are not a direct argument for the differences in the context memory. On the other hand, however, the patterns of memory functioning found in studies using FTT and the context effects observed in DD reading performance suggest that there can be a dyslexia-specific impairment in context recollection that is worth investigating within DRT. The presented study investigates verbal and non-verbal memory functioning in DD with the use of this theoretical approach and multinomial modeling analysis.

### Study hypotheses

Based on the available literature and theories of memory functioning in dyslexia we can formulate the following hypotheses:

1. Target recollection is impaired in DD in comparison with TD in the case of verbal material.
2. Target recollection is impaired in DD in comparison with TD in the case of visual material. We base this hypothesis on the findings that show a significant deficit in visual memory in DD (e.g., Smith-Spark et al., 2003; Smith-Spark and Fisk, 2007; Bacon et al., 2013). We assume that there are processes that are responsible for both verbal memory and visual memory impairment. The use of multinomial modeling may enable us to pinpoint the possible sources of inconsistencies in previous findings on the presence of visual memory deficits in DD.
3. Context recollection for semantic operations on verbal material is impaired in DD in comparison with TD.
4. Context recollection for perceptual operations on verbal material is enhanced in DD in comparison with TD.
5. Target recollection for semantically processed verbal material is weaker in comparison to target recollection for perceptually processed verbal material in the DD group.
6. Context recollection for semantic operations on verbal material is weaker in comparison to context recollection for perceptual operations on verbal material in the DD group.
7. There is a stronger relationship between verbal and visual memory in DD as compared to TD. We base this hypothesis on experimental findings and theories (e.g., the magnocellular hypothesis) suggesting that there is a specific connection between visual and verbal processing in the DD group (e.g., Vellutino, 1979; Snowling, 2019; Stein, 2019). As there are findings indicating a greater effect of visual cues or visual processing on reading and reasoning in the DD group compared to the TD group, we assume by analogy that there would also be a stronger connection between verbal and visual memory in DD than in TD.

## Materials and methods

### Participants

The subjects were 82 young adults (aged 18–31 years) who were compensated for their participation in the study with gift cards valued at PLN 40 (*ca*. €9). The participants were recruited to one of two groups: (1) with diagnosed developmental dyslexia; (2) typically developing. Groups were of equal size (*N* = 41). Due to the specific and difficult to recruit DD group, the number of participants was limited to the obtainable sample. Sensitivity analyses conducted (using G*Power 3 software: Faul et al., 2007) separately for multinomial models for verbal and pictorial material ensured a high test power: 1–β = 0.80. With the total number of responses across participants ranging from 4,920 to 6,888, small effect sizes (*w*) ranging from 0.040 to 0.034 were detectable, for verbal or pictorial conditions, respectively. If we translate these *w* effect size parameters into the minimal differences between model parameters, for the context recollection parameters, we obtain differences of 0.15/0.12 (for verbal/pictorial material, respectively); for the target recollection parameters, we obtain a difference of 0.17/0.16; for the familiarity parameters, we obtain differences of 0.37/ 0.38; and finally, for the response bias parameters, we obtain differences of 0.05/0.03 (computed with the post-hoc power analysis option in multiTree; Moshagen, 2010).

The initial recruitment to the DD and TD groups was conducted based on a diagnosis of developmental dyslexia (or lack thereof) made during the period of school education. In the second stage of recruitment, a Polish adaptation of the Revised Adult Dyslexia Checklist (Vinegrad, 1994; Bogdanowicz and Krasowicz-Kupis, 2000) was used. This short questionnaire consists of 20 items with a “yes/no” response scale and can be applied as a screening test for dyslexic adults. The questionnaire items consist of questions about typical dyslexia symptoms and problems that may occur during adulthood, for example: “Do you take longer than you should to read a page of a book?” (item 4) or “Do you find forms difficult and confusing?” (item 18). Potential participants with and without a dyslexia diagnosis were classified for the experiment only if their overall number of “yes” answers was higher or equal to 9 (or 6 counting the strongest items) for the former, or lower than 9 (or 6 counting the strongest items) for the latter. Those who did not meet this criterion were not included in the experiment. Of the 95 participants who applied for the experiment, three did not respond to the email to schedule an experiment, and 10 were not classified based on their questionnaire score (seven of them without a dyslexia diagnosis and three of them with a dyslexia diagnosis).

### Materials

In the presented study, two types of materials were used: verbal and pictorial. The verbal condition material consisted of: (1) 36 word triads of semantically and phonologically related items to the target (e.g., *sofa*—*couch*—*soda* or *sword*—*blade*—*sworn*: Obidziński and Nieznański, 2017); (2) 18 pairs of words unrelated either on the semantic or the phonological level (target-unrelated: e.g., *fisher*—*game*); and (3) 24 words used as unrelated distractors in the test phase. Three pairs of words were added to the study list as a primacy buffer, and three other pairs as a recency buffer.

The pictorial condition material consisted of: (1) 48 picture triads of semantically related, similar in appearance items to the target (e.g., pictures of a ring—earring—donut); (2) 24 pairs of pictures unrelated either on the semantic or the visual level; and (3) 36 pictures used as unrelated distractors in the test phase. Three pairs of pictures were added to the study list as a primacy buffer, and three other pairs as a recency buffer. The drawings used as targets were larger (300 × 300 pixels) than the images used as the referent stimuli (200 × 200 pixels). All the pictures presented colored objects against a white square background. Pictures were taken from the De Groot et al. (2016) set and from the MultiPic database (Duñabeitia et al., 2018: for a detailed description of the material selection procedure, see Nieznański, 2020).

We used more stimuli in the pictorial memory condition than in the verbal condition to make conditions similar in difficulty, since memory for pictures is known to be better than for words (e.g., Shepard, 1967; Paivio et al., 1968; Paivio and Csapo, 1973). All the verbal items can be found on the Internet repository of the study.^1^

### Procedure

The procedure and design of the conducted experiment are based on a modified version of the dual recollection paradigm (see Brainerd et al., 2015; Nieznański, 2020). The memory experiment consisted of two separate tasks, the verbal materials and then the pictorial materials. To control the potential effects of task order on the collected data, the order of the verbal vs. pictorial tasks was counterbalanced across the participants: half of the participants started the experiment with a verbal task followed by a pictorial task, and the second half started the experiment with a pictorial task followed by a verbal task.

The overall procedure of the two tasks was practically identical. The experiment started with the study phase during which the participants had to memorize the study material. The presentation duration was 5 s per item and, during this period of time, the participants were asked one of the two “yes/no” questions (Polish “tak/nie”) that required a given stimuli to be compared with an adjacent word/picture. Participants were asked to answer the question during the presentation of a stimulus, by pressing *T* (for “yes”) or *N* (for “no”) on the computer’s keyboard. In the verbal condition, the participants were asked “Does it sound similar to X?” or “Does it share a meaning with X?.” Across both conditions, the participants were presented with 36 pairs of similar stimuli and 18 that were not similar (in sum, 60 words were studied during this phase of the memory experiment including six buffers). The words from the unrelated pairs were not used at the memory test sessions. In the pictorial condition, the participants were asked “Does it look similar to X?” or “Does it share a meaning with X?” The participants were presented across both conditions with 48 pairs of similar stimuli and 24 that were not similar (in sum, 78 pictures were studied during this phase of the memory experiment including six buffers). The pictures from the unrelated pairs were not used in the test. There were also six buffer stimuli, three at the beginning and three at the end of the study list. Three of the buffer stimuli were presented with a question about the perceptual similarity, and three with a question about the semantic similarity. They were used to reduce the serial-position effect.

In the test phase of the memory experiment, the participants were presented with old and new stimuli. Half of the targets were presented with a question about perceptual similarity, and the other half were presented with a question about semantic similarity. Thus, there were three types of test stimuli. For each presented stimulus, the participants were asked to answer “yes” or “no” to the memory probe. There were three types of memory probes used in the test: (1) “Was it presented with the question about the similarity in sound/appearance?”; (2) “Was it presented with the question about the similarity of meaning?”; and (3) “Was it presented with any question? (either about the similarity in sound/appearance or meaning).” The responses were self-paced.

Words were presented in Times New Roman font, 32-point size. The instructions were presented on computer screens and the participants started the task once they became familiar with the instructions. The experiment was conducted with the use of the E-Prime program 2.0 (Psychology Software Tools, Pittsburgh, PA, United States) on the same model of notebooks, with 15-inch monitors with a refresh rate of 60 Hz and a resolution of 1,366 × 768.

In the study phase, 18 words or 24 images from related pairs were presented with a perceptual similarity orienting question, and 18 words or 24 images were presented with a semantic similarity orienting question. The same targets, along with unrelated distractors (24 words or 36 images), were presented in the test phase. They were presented with the probe referring to the kind of orienting question used during the study phase. Therefore there were 36 old and 24 new items presented in the verbal memory test, and 48 old and 36 new items presented in the visual memory test. Stimuli were presented along with the test probe and remained on the screen until participants gave their answer. Each stimulus was presented with only one of the three test probes for a given participant. Test probe—stimulus pairings varied randomly among participants.

The number of missing and incorrect answers to orienting questions was compared between groups to control for potential differences caused by limited study time. Since the numbers were very low and not significantly different between DDs and TDs, we decided not to exclude targets with failures on orienting questions from the analysis.

### Multinomial model

The multinomial dual-recollection model (Brainerd et al., 2015)─the overall theoretical background of which was described in the introduction section of this paper─was originally constructed for the experiment with two sources of information (e.g., List 1 and List 2), and one type of experimental material (words). However, in the present study a modified version of this model, adapted for the memory for orienting question task, was used (Nieznański, 2020). In this version, the context of the target is the type of question asked when presented during the study phase. The modified version incorporates two types of material: verbal and pictorial. This not only allows for a broader examination of the context and target memory but also the comparison of corresponding parameters for different presentation modes.

Because of these changes, the model has been extended and the number of model trees and parameters has been doubled. However, because the new parameters mirror each other, each parameter will be described only once, noting that it can be a parameter of the verbal or the pictorial experimental condition. The parameter descriptions are presented in Table 1. In the present study, the model was fitted for data aggregated across participants.

**Table 1:** Parameters of the multinomial dual-recollection model used in Experiment 1.

Parameter	Description
*CP*	Context recollection for the perceptual orienting question—the probability that a target provokes the conscious reinstatement of some of the contextual details that accompanied its presentation with a question about the perceptual similarity during the study phase.
*CS*	Context recollection for the semantic orienting question—the probability that a target provokes the conscious reinstatement of some of the contextual details that accompanied its presentation with a question about the semantic similarity during the study phase.
*FP*	Familiarity for the perceptually processed item—the probability that a target presented with a perceptual orienting question provokes a sufficiently high level of familiarity that subjects accept it with a probe of “Was it presented with any question?”
*FS*	Familiarity for the semantically processed item—the probability that a target presented with a semantic orienting question provokes a sufficiently high level of familiarity that subjects accept it with a probe of “Was it presented with any question?”
*TP*	Target recollection for the perceptually processed item—the probability that a target accompanied by a question about a perceptual similarity on the study list provokes the conscious reinstatement of its presentation.
*TS*	Target recollection for the semantically processed item—the probability that a target accompanied by a question about the semantic similarity on the study list provokes the conscious reinstatement of its presentation.
*b*	Response bias for specific probes—the probability of an answer that is biased toward recognizing an item as old when the target provokes an insufficient level of familiarity and there is a lack of conscious reinstatement when the probe asks about a specific context.
*bPS*	Response bias for the “Was it presented with any question?” probes—the probability of an answer that is biased toward recognizing an item as old when the target provokes an insufficient level of familiarity and there is a lack of conscious reinstatement when the probe asks about any context.

Each of the parameters occurs in separate models for verbal and pictorial stimuli. In the following, this is indicated by adding the Index “VERBAL” or “PICTORIAL” to the respective parameter.

**Figure 1:**
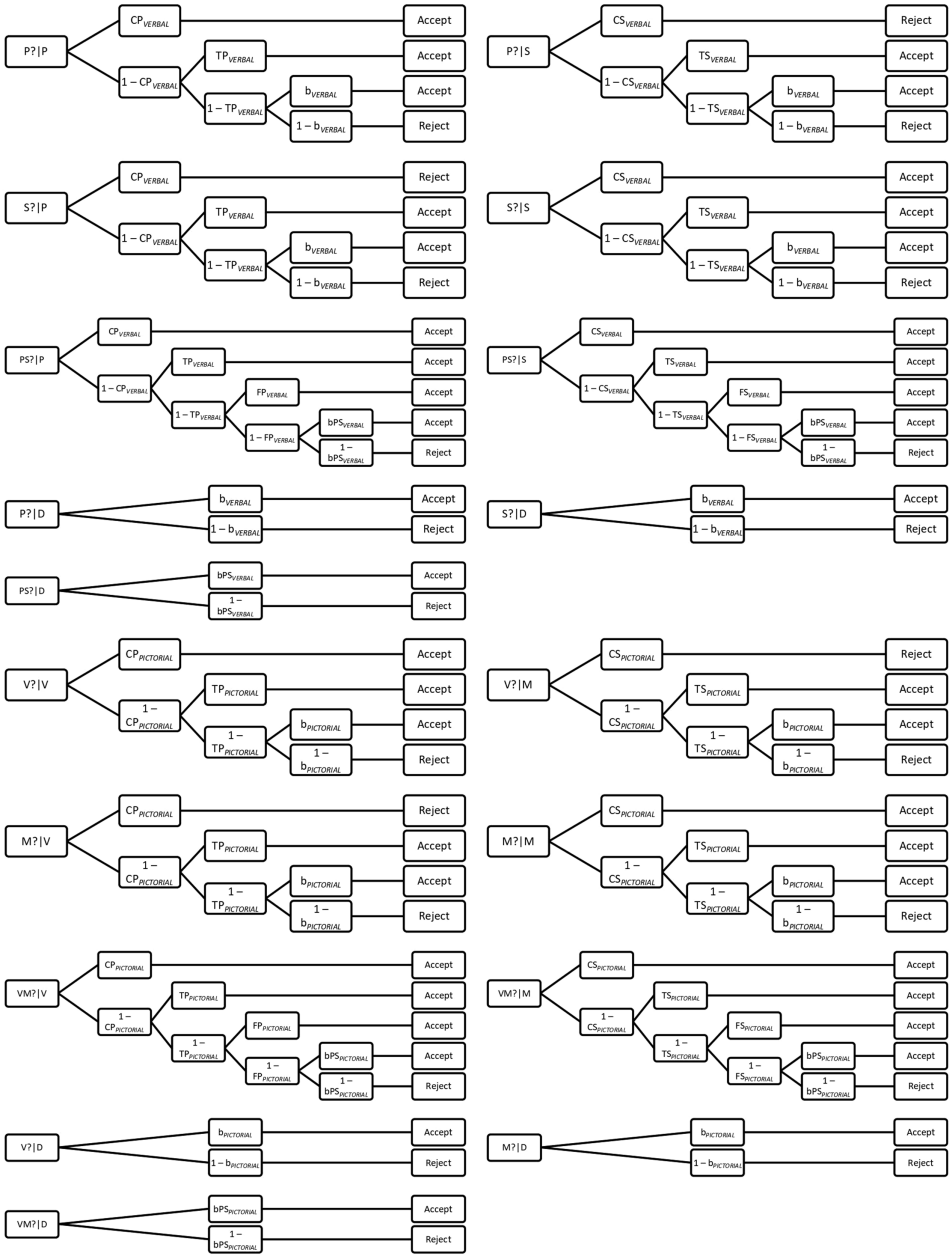
Dual recollection multinomial tree model for verbal and pictorial conditions (Brainerd et al., 2015; Nieznański, 2020). P/V—perceptual context for targets in verbal (Phonological)/visual (Visual) conditions; S/M—semantic context for targets in verbal (Semantic)/visual (Meaning) conditions; D—distractor; P?/V?—perceptual probe presented during test in verbal/visual conditions; S?/M?—semantic probe presented during test in verbal/visual conditions; and PS?/VM?—“perceptual or semantic” probe presented during test in verbal/visual conditions.

Figure 1 above presents trees of the multinomial model for all types of test stimuli, for both verbal and pictorial conditions. The model consists of 18 trees, nine for verbal and nine for pictorial material, and describes all possible routes of cognitive processes assumed in the theoretical framework, leading to one of two possible answers (acceptance or rejection) in reaction to the test probe. In the presented model, there are separate trees for all possible combinations of test stimuli and test probes, for both verbal and pictorial material. The roots of a given tree indicate the type of stimulus and probe, while the leaves specify response categories. In turn, tree branches represent multinomial model equations that are used to estimate values of model parameters, therefore, estimating the probability of a given cognitive process. As there are two degrees of freedom (in each model: for verbal and pictorial memory) for the goodness-of-fit test, the model can be tested.

As can be seen in Figure 1, when a target source is congruent with the question probe (e.g., P?|P), the target cues are accepted if the context recollection (e.g., *CP*) or the target recollection (e.g., *TP*) is successful. If neither is successful, the response bias (*b*) can produce acceptance. On the other hand, when a target source is incongruent with the question probe (e.g., S?|P), the target cues are rejected if context recollection is successful. However, they are accepted if context recollection fails (1-*CP*) but target recollection (*TS*) is successful. Furthermore, acceptance responses may also be produced by the response bias (*b*). Finally, on probes with the perceptual or semantic (PorS?) question (e.g., PS?|P), the participants accept the probe if the context recollection, target recollection, or familiarity (*FP*) are successful. Only if all of these retrieval processes fail can the response bias (*bPS*) produce acceptance. For distractors, only the response bias (*b* for P? and S? probe questions, and the *bPS* for PorS? probe question) can produce acceptance (*cf*. Brainerd et al., 2015; Nieznański, 2020).

## Data analysis

In the presented analysis the dependent variables were: (1) acceptance probabilities given a particular type of stimulus and test question, and (2) the parameters of the multinomial model measuring the contribution of context recollection, target recollection, familiarity, and guessing bias to memory task performance. Importantly, we treated each comparison of a parameter between the DD and TD groups as a test for a separate hypothesis, so there was no need to make adjustments for multiple comparisons.

### Standard analyses

Because of the non-normal distribution of all the variables, non-parametrical statistical measures of difference will be used in the presented analysis. First, we report an analysis of the proportions of acceptance in the memory test for the verbal condition, followed by the pictorial condition, and finally an analysis is carried out of the correlation of these proportions between the conditions.

#### Words

The descriptive statistics for the proportions of acceptance in the memory test for dyslexics, typically developing participants, and the whole group are presented in Table 2. The analyses are not related to a single specific hypothesis, as they are stated for the parameters of the multinomial model. However, standard analyses of verbal memory performance can be connected more broadly to hypotheses 1, 3, and 4—as the proportions of correct responses can indicate, for example, that context memory is impaired for a given type of context.

**Table 2:** Descriptive statistics for the probability of “yes” answers for different groups and test probes for the verbal material and differences test results.

	Participants with dyslexia *N* = 41		Typically developing participants *N* = 41		Mann–Whitney’s U test	
	*M*	*SD*	*M*	*SD*	*Z_corrected_*	*p*
*PP*	0.594	0.264	0.594	0.253	0.085	0.932
*PPS*	0.594	0.239	0.622	0.242	−0.450	0.653
*PS*	0.297	0.228	0.224	0.238	1.620	0.105
*NP*	0.125	0.166	0.117	0.144	−0.005	0.996
*NPS*	0.070	0.098	0.059	0.085	0.810	0.418
*NS*	0.101	0.168	0.080	0.112	−0.540	0.589
*SP*	0.333	0.271	0.281	0.237	0.848	0.396
*SPS*	0.833	0.186	0.866	0.155	−0.631	0.528
*SS*	0.785	0.187	0.894	0.143	−2.861	0.004

PP, acceptance probability for words encoded in the phonological context, when asked about the presentation in the phonological context; PPS, acceptance probability for words encoded in the phonological context when asked about the presentation in any context; PS, acceptance probability for words encoded in the phonological context when asked about the presentation in semantic context; NP, acceptance probability for new words when asked about the presentation in the phonological context; NPS, acceptance probability for new words when asked about the presentation in any context; NS, acceptance probability for new words when asked about the presentation in the semantic context; SP, acceptance probability for words encoded in the semantic context when asked about the presentation in the phonological context; SPS, acceptance probability for words encoded in the semantic context when asked about the presentation in any context; and SS, acceptance probability for words encoded in the semantic context when asked about the presentation in the semantic context.

The U-Mann–Whitney test was used for between-group comparisons. The results showed only one significant difference in the acceptance probability for words encoded in the semantic context when asked about the presentation in the semantic context (SS) condition: *Z*_corrected_ = 2.861, *p* = 0.004, *r* = 0.447. Therefore, there is a significantly lower probability of a correct “yes” answer in this condition for participants with dyslexia. This result is in line with hypothesis 3: target recollection is impaired in DD in comparison with TD in the case of visual material. All the other differences were not significant.

#### Pictorial material

The descriptive statistics for the proportions of acceptance for participants with dyslexia, typically developing, and the whole group are presented in Table 3. Standard analysis of visual memory performance can be used to indirectly test hypothesis 2 about target recollection impairment in DD in comparison with TD in the case of visual material.

**Table 3:** Descriptive statistics for the probability of a “yes” answer in different test conditions for the pictorial material and differences test results.

	Participants with dyslexia *N* = 41		Typically developing participants *N* = 41		Mann–Whitney’s U test	
	*M*	*SD*	*M*	*SD*	*Z_corrected_*	*p*
*VV*	0.893	0.107	0.869	0.183	−0.129	0.898
*VVM*	0.930	0.125	0.860	0.166	2.681	0.007
*VM*	0.217	0.224	0.180	0.192	0.603	0.547
*NV*	0.059	0.097	0.041	0.090	1.153	0.249
*NVM*	0.069	0.093	0.035	0.083	2.547	0.011
*NM*	0.043	0.088	0.031	0.081	0.982	0.326
*MV*	0.235	0.241	0.220	0.214	0.095	0.924
*MVM*	0.820	0.161	0.845	0.160	−0.788	0.431
*MM*	0.762	0.203	0.756	0.185	0.324	0.746

VV, acceptance probability for the pictures encoded in the visual context when asked about the presentation in the visual context; VVM, acceptance probability for the pictures encoded in the visual context when asked about the presentation in any context; VM, acceptance probability for the pictures encoded in the visual context when asked about the presentation in the semantic context; NV, acceptance probability for new pictures when asked about the presentation in the visual context; NVM, acceptance probability for new pictures when asked about the presentation in any context; NM, acceptance probability for new pictures when asked about the presentation in the semantic context; SV, acceptance probability for pictures encoded in the semantic context when asked about the presentation in the visual context; SVM, acceptance probability for the pictures encoded in the semantic context when asked about the presentation in any context; and SM, acceptance probability for the pictures encoded in the semantic context when asked about the presentation in the semantic context.

Once again, the U-Mann–Whitney test was used. The results show two significant differences: in the acceptance probability for pictures encoded in the visual context when asked about the presentation in any context (VVM: *Z*_corrected_ = 2.681, *p* = 0.007, *r* = 0.419), and in the acceptance probability for new pictures when asked about the presentation in any context (NVM: *Z*_corrected_ = 2.547, *p* = 0.011, *r* = 0.398). For both variables, a higher probability of a “yes” answer was observed in the dyslexia group. All the other differences were not significant. These results do not support hypothesis 2.

#### Relations between memory of words and images.

The correlations between the corresponding measures of the verbal and pictorial memory tests were measured using Spearman’s *ρ* coefficient. The results of these analyses are presented in Table 4.

**Table 4:** Spearman’s rho correlation coefficients between the probabilities of a “yes” answer in the verbal and pictorial conditions separately for the dyslexic (D) and typically developing (T) groups.

	*PP*-*VV*	*PPS*-*VVM*	*PS*-*VM*	*NP*-*NV*	*NPS*-*NVM*	*NS*-*NM*	*SP*-*MV*	*SPS*-*MVM*	*SS*-*MM*
D	0.524***	0.054	0.455**	0.297	0.244	0.418**	0.279	0.122	−0.080
T	0.094	0.295	0.219	0.228	0.215	0.296	0.363*	−0.123	0.140

^*^<0.05, ^**^<0.01, and ^***^<0.001.

Differences between the groups were observed in both the significance of correlations and their strength. In the dyslexic group, we observed significant correlations for the PP-VV, PS-VM, and NS-NM parameters. In a typically developing group, we observed significant correlations only for SP-MV parameters. All the significant correlations in the DD group are moderate and one significant correlation in the TD is weak. There are no correlations between verbal and pictorial memory measures that are significant in both groups; the correlation in the dyslexic group is stronger than in the typically developing group. The difference in correlations between groups was tested for variables where there was at least one significant correlation in either the DD or TD group. Only one of them—the correlation between PP and VV—was found to be significantly different between DD and TD. It was higher in the DD group: *t*(78) = 2.125, *p* = 0.034, and only in this group did it reach a significant level (for test description and equation see Cohen et al., 2003; Revelle and Revelle, 2022, pp. 318–322).

### Multinomial modeling analyses

To test differences in parameters between groups, we start with the combined baseline MPT model created for both groups, then we put an equality constraint on the parameters (e.g., CVverbal for DD group = CVverbal for TD group) and check the significance of the change in the model fit after imposing this constraint. All computations were carried out with the multiTree computer program (Moshagen, 2010).

An analysis of the goodness-of-fit for the used models showed that they fit well to both verbal [*G^2^*(2) = 3.356, *p* = 0.187] and pictorial [*G^2^*(2) = 2.210, *p* = 0.331] stimuli. Therefore, further analyses are conducted to compare the DD and the TD groups and to analyze the differences within the groups. To test our hypothesis we planned comparisons of the following parameters between DD and TD: *TP*_VERBAL_, *TS*_VERBAL_, *TP*_PICTORIAL_, *TS*_PICTORIAL_, *CP*_VERBAL_, and *CS*_VERBAL_. In the case of within-group comparisons, we planned *CP*_VERBAL_ vs. *CS*_VERBAL_ and *TP*_VERBAL_ vs. *TS*_VERBAL_ comparisons, based on our hypotheses. Moreover, to further explore the differences between the DD and the TD groups, we also conducted comparisons for the other parameters of the model and in pairs of corresponding parameters. Table 5 presents the estimates of the model parameters for both the verbal and the pictorial material and the results of the comparisons between the groups.

**Table 5:** Parameter estimates and their standard errors for the dual processing multinomial model with the verbal and pictorial memory in the context of the perceptual and semantic similarity and the results of G^2^ tests of between-groups comparisons.

Parameter	DD		TD		Comparison
	*p*	*SE*	*p*	*SE*	
*CP*_VERBAL_	0.297	0.043	0.370	0.041	
*CS*_VERBAL_	0.451	0.040	0.602	0.035	*ΔG*^2^(1) = 8.822, *p* = 0.003
*FP*_VERBAL_	0.050	0.106	0.110	0.101	
*FS*_VERBAL_	0.265	0.139	0.000	0.224	
*TP*_VERBAL_	0.337	0.037	0.284	0.037	
*TS*_VERBAL_	0.550	0.042	0.673	0.045	Δ*G*^2^(1) = 4.643, *p* = 0.031
*b*_VERBAL_	0.128	0.013	0.099	0.012	
*bPS*_VERBAL_	0.082	0.015	0.058	0.013	
*CP*_PICTORIAL_	0.677	0.028	0.685	0.028	
*CS*_PICTORIAL_	0.527	0.033	0.537	0.033	
*FP*_PICTORIAL_	0.330	0.172	0.000	0.191	
*FS*_PICTORIAL_	0.229	0.119	0.363	0.103	
*TP*_PICTORIAL_	0.652	0.045	0.554	0.046	
*TS*_PICTORIAL_	0.470	0.037	0.454	0.037	
*b*_PICTORIAL_	0.051	0.007	0.036	0.006	
*bPS*_PICTORIAL_	0.069	0.011	0.034	0.008	Δ*G*^2^(1) = 6.156, *p* = 0.01

Three statistically significant differences were observed between the participants with and without developmental dyslexia: (1) for the context recollection parameter in the semantic context of the verbal condition (*CS*_VERBAL_); (2) for the target recollection parameter in the semantic context of the verbal condition (*TS*_VERBAL_), and (3) and for the response bias parameter for the “perceptual or semantic?” probes in pictorial condition (*bPS*_PICTORIAL_).

Finally, Table 6 presents the results of within-group comparisons conducted between the memory parameters for the perceptual vs. the semantic conditions, separately for the DD and the TD groups. As shown in Table 6, most of the differences are significant in both groups. Significant differences were not found between the familiarity parameters in the DD and the target recollection parameters in the TD; however, there was a statistical tendency in the latter. In both groups, there was no difference in the familiarity parameters in the verbal condition. In one case of the target recollection parameter for the pictorial material, we only observed a statistical tendency in the TD but a significant difference in the dyslexic group.

**Table 6:** Results of within-group comparisons between the model parameters.

	DD		TD	
*Parameters*	Δ*G*^2^(1)	*p*	Δ*G*^2^(1)	*p*
*CP*_VERBAL_ vs. *CS*_VERBAL_	7.244	0.007	20.373	< 0.001
*FP*_VERBAL_ vs. *FS*_VERBAL_	1.412	0.235	0.699	0.403
*TP*_VERBAL_ vs. *TS*_VERBAL_	15.780	< 0.001	43.717	< 0.001
*CP*_PICTORIAL_ vs. *CS*_PICTORIAL_	11.691	< 0.001	12.195	< 0.001
*FP*_PICTORIAL_ vs. *FS*_PICTORIAL_	0.222	0.638	4.044	0.044
*TP*_PICTORIAL_ vs. *TS*_PICTORIAL_	9.556	0.002	3.189	0.074

## Discussion

Although the results of the conjoint recognition memory experiment did not demonstrate systematic and broad differences between the DD and TD groups, some significant differences were confirmed by the analyses. Referring directly to the hypotheses we set, the results we obtained speak in favor of DD impairment of target recollection and semantic context recollection for verbal material, as well as a stronger relationship between verbal and visual memory in DD, compared to TD (hypotheses 1, 3, and 7). On the other hand, impairment of target recollection for visual memory, enhancement of pictorial context memory, and the notion of weaker target and context memory for items with a semantic context in comparison to the perceptual context in DD (hypotheses 2, 4, 5, and 6) are not supported by the results of the analyses. In the case of hypothesis 6, however, it can be noted that the difference between the context recollection parameters for verbal material is smaller in the DD than in the TD group. Nevertheless, this difference is not statistically significant. In this part of the article we will first discuss the results of the standard analysis, then the results of the modeling, and finally the overall relevance of the observed differences to better understand the specific patterns of memory functioning in individuals with developmental dyslexia.

### Discussion of the results of standard statistical analyses

We observed only one significant difference in the acceptance probability for words encoded in the semantic context when asked about their presentation, which turned out to be significantly higher in the TD group. The size of the observed significant effect was moderate. Thus, as we expected, we observed that memory for words is weaker in the developmental dyslexia group than in the TD when context refers to the orienting task about semantic meaning.

It should be noted, however, that the difference for the semantic context and the lack of difference in the phonological context (sound of words) does not seem to be consistent with some studies, including our own (Obidziński and Nieznański, 2017), investigating the connection between dyslexia and the different types of representation and processing: formal (verbatim) and semantical (gist). Taking into account previous studies (e.g., Snowling et al., 1994; Nation and Snowling, 1998; Vellutino et al., 2004; Miles et al., 2006; Taroyan and Nicolson, 2009; Obidziński and Nieznański, 2017) we would rather expect the opposite pattern: no differences in the semantic context, but a weaker memory of the phonological context.

An analysis of the experimental methodology (Brainerd et al., 2015; Nieznański, 2020) could give a possible explanation for the observed discrepancy. Because of the applied material presentation method, there is a need for greater language processing in the study phase of the present experiment than in a standard memory experiment or in context memory procedures where only one or two words are typically presented at the same time (e.g., Kramer et al., 2000; Nieznański, 2013). Not only do the study items and the stimuli being compared need to be processed verbally, but the instructions also require adaptation to indicate how such a comparison should be made. The fact that memorizing material while reading is resource intensive can be illustrated by the Reading Span task (e.g., Daneman and Carpenter, 1980; Friedman and Miyake, 2004) in which the participant has to do a comprehension task while also memorizing the final word of each sentence. Therefore, taking into account that semantic processing is already involved not only in item but also in other information processing (*cf*. Nation and Snowling, 1998; Vellutino et al., 2004), we argue that this is a possible source for the reversal of the pattern of results. Semantic information helps with the overall memorization of an item but decreases the cognitive resources for context memorization when it is semantic, although it has no effect when the context is phonological.

In the pictorial memory experiment, we observed two significant differences. First, the acceptance probability for the pictures encoded in the visual context when asked about the presentation in any context is higher in the dyslexia group. Second, the acceptance probability for new pictures when asked about the presentation in any context is higher in the dyslexia group. The size of both the observed significant effects is moderate. Therefore, we found better scores in the recognition of old items in the dyslexic group, as well as a higher amount of false alarm errors. The observed pattern is interesting because it shows the difference not only in verbal but also in pictorial memory. Overall, it is consistent with some findings (e.g., Smith-Spark and Fisk, 2007; Menghini et al., 2011; Talepasand et al., 2018) but inconsistent with others showing no decline in the pictorial memory or the overall visual ability (see McManus et al., 2010; Snowling, 2019). This pattern also suggests that the differences in memory functioning in dyslexia are not consistent between the different modalities. Moreover, the findings of the presented study suggest that pictorial memory may be viewed as a compensatory mechanism of dyslexia rather than its pathomechanism. Previous research has mostly, but not always (e.g., Bacon and Handley, 2014), theoretically analyzed and investigated the latter concept (*cf*. Menghini et al., 2011; Talepasand et al., 2018).

However, these differences are only significant when questions are asked about any source. In terms of both the visual and the semantic similarity, the observed differences are not significant. Nevertheless, numerically, the proportion of “yes” responses is higher in the DD group in almost all (except one) conditions. Taking this into account, one possible explanation is that we observe a specific “response bias” (Rotello and Macmillan, 2007) in the dyslexic group, where adults with developmental dyslexia are more likely to answer “yes” in most situations in the pictorial memory task. The results presented would be similar and consistent with the interaction observed in the Tuomainen (2015) study, where memory for nonspeech sounds was more liberal (more “yes” answers) than for the speech sounds condition only in the dyslexic group. Nevertheless, this explanation does not consider the fact that only two out of all the parameters were significantly different and that the difference in these two situations is greater for hits than for false alarms. This suggests that even if the bias hypothesis is true, other variables may affect the pictorial memory of adults with dyslexia.

Finally, we will now discuss the results of the correlation analysis conducted on the corresponding memory parameters from both experiments. A separate analysis was conducted for the DD and the TD groups to search for possible differences in the correlation pattern that may be relevant to further discussion. The analysis does indeed show different patterns of correlation between the memory parameters. There is one significant difference between the groups, where memory of verbal material correlates with memory of visual material only in the DD group—and this correlation is of moderate strength, while in the TD group the correlation coefficient is close to 0. Furthermore, analysis revealed that there is no significant correlation that is consistent between the groups. The significant correlations for the dyslexic group are correlations for old items presented with a question about the perceptual similarity (when the probe asks about a specific context: either perceptual or semantic) and for the parameters measuring the probability of a “yes” answer when a new item is presented with a probe about the semantic context. On the other hand, the only significant correlation for the TD is the correlation between the measures of memory for the old items presented with a semantic question (when the probe asks about the perceptual similarity). Thus, there is a discrepancy between the contexts in which the correlations are significant for the studied groups. This may suggest that there is a specific relation between visual and verbal processing in dyslexia (Bacon and Handley, 2014; Snowling, 2019).

Importantly, the indicated discrepancy seems to be in line with the research findings and the theoretical conceptualizations that point to the importance of visual processing in dyslexia and its relation to verbal processes in this learning disorder, which affect different processes, such as reading and reasoning (e.g., Bacon and Handley, 2014; Snowling, 2019). For example, some studies show that dyslexic persons use visual processes more than verbal processes in reading compared to the typically developing group (*cf*. De Luca et al., 2002). Others show that dyslexic verbal memory (memory for letters)─the impairment of which is well documented─can improve when more visual processing (tracing the letters) is involved, while no improvement is observed in the TD in the same situation (*cf*. Vellutino, 1979; Snowling, 2019). Also, some pathomechanism theories, such as the magnocellular deficit theory (e.g., Stein, 2019), emphasize the role of visual processing in the dyslexic reading process. Therefore, the visual processing of verbal stimuli seems to be more important in the verbal processing of the dyslexic than the typically developing group. This only results in significant correlations between verbal and visual memory measures in the perceptual condition for participants with dyslexia. As for the discrepancy in the correlation in the semantic context─the fact that we only observed a significant correlation in the TD group can be connected with the experimental methodology, as described in the discussion of the results of other analyses above.

### Discussion of the results of multinomial modeling analyses

The analysis conducted with the use of the multinomial processing tree model for dual-recollection theory shows both significant differences between the studied groups and significant differences between the model parameters for the different types of context within the studied groups. First, we will discuss the between-group comparisons. In the parameters for the verbal memory experiment, we observed three statistically significant differences between dyslexic and typically developing groups that show a selective—affecting only one of two parameters—difference in both the context and the target memory. In both these cases, the model estimates a lower probability of recollection in participants with dyslexia.

An impairment of target recollection is an important finding because it corresponds to the recollection rejection deficit we found in the study of verbatim and gist memory in dyslexia (Obidziński and Nieznański, 2017). Therefore, it shows a possible memory-based pathomechanism of developmental dyslexia. As memory is important for all language processes, including reading and writing (*cf*. Everett, 2012; Carreteiro and Figueira, 2017), an impairment of this particular memory process can rather be clearly linked to the type of problem and the language errors observed in developmental dyslexia (e.g., American Psychiatric Association, 2013; Snowling, 2019). On the other hand, context recollection impairment (which corresponds to either the gist trace retrieval according to older FTT, or the context trace retrieval in the current theoretical model), could be explained as in the discussion of the standard analyses. The method used is demanding for verbal processing during the study phase of the experiment and because of context facilitation (e.g., Nation and Snowling, 1998), we observed its impairment but only in the case of the parameter for the semantic context, which is the most loaded by verbal processing.

In the pictorial material condition, only one significant difference was found in the model parameters between the groups. The parameter of the response bias when the probe asks about either the visual or the semantic context is higher in the dyslexic group. Therefore, our interpretation about bias being the source of the difference observed in the standard analysis discussed earlier gains further support thanks to the modeling analysis. Moreover, the bias parameter is significantly different only for the “either visual or semantic” probe type, thus, it nicely corresponds with the observed significant differences on the standard statistics level. The fact that the bias difference is significant only in pictorial memory and its direction is that more “yes” answers are observed is also consistent with the findings for the memory of sounds (*cf*. Tuomainen, 2015).

Finally, a within-group analysis of the memory parameters will be discussed. In the case of most comparisons, there are significant differences in parameters between the perceptual and the semantic conditions. The context recollection parameters in both groups are higher for the semantic than for the perceptual level of processing in verbal memory, but lower for pictorial memory. The familiarity parameter difference is significant only in the case of pictorial memory in the TD, where the semantic familiarity parameter is higher than the perceptual one. Finally, in the case of target recollection, we found the same pattern as in the context recollection parameters, where the verbal parameters are higher in the semantic condition and the pictorial in the perceptual condition, for both groups. Therefore, the results show the same overall pattern of relations between memory parameters and type of context in both studied groups. Furthermore, this pattern replicates the findings of the previous study (Nieznański, 2020) using the same experimental paradigm on a sample from the general population. Thus, in the case of the relationship between the type of material and the context, adults with dyslexia did not show any specific pattern of memory functioning that would be different from their typically developing peers.

### General discussion

We now turn to a more general interpretation of our results. Firstly, memory impairments in DD seem to be present in the case of both target and context recollection. Therefore, problems with recollection memory are not limited to target-specific information, but also involve contextual information about the cognitive operations performed during the learning phase. Concerning DD, problems with the memory of verbal information do not seem to be limited to just item memory, but also its context, showing a layer of memory dysfunction not observed in previous studies. Furthermore, this fact suggests that problems with item memory could be further amplified by the problems with context memory. In everyday situations, individuals with DD may not only have difficulty with retrieving some important information but also the context in which this information was acquired, which in turn makes the process of remembering even harder. It could be especially problematic in higher education situations or the workplace when the technical and highly formalized language of written information makes it difficult for individuals with DD to process and remember it (*cf*. Obidziński and Nieznański, 2017; Snowling, 2019).

However, it needs to be highlighted that these problems are observed only in the case of a semantic context. As proposed earlier, this pattern of results could be connected with the fact that in DD semantic and context information are used as the compensatory mechanism of the reading process (Nation and Snowling, 1998; Vellutino et al., 2004). Therefore, because individuals with DD have difficulties reading and need semantic context to facilitate reading, their memory for semantic contexts is impaired in the experimental procedure, which is more demanding for reading than the typical one. Therefore, significant differences are observed only in the condition in which semantic and context processing are most loaded. Nevertheless, this fact did not change the described implications, but rather made the experimental procedure even more similar to an everyday situation. When reading documentation, a textbook, an article, or any other type of written information, individuals with DD are more likely than their TD peers to use semantic and contextual facilitation, making these processes more loaded. That is why, in everyday situations, we should expect similar problems with target and context memory in adult individuals with dyslexia. This fact should prompt reflection on methods to mitigate the effects of difficulties in processing written information that could be used to help people with DD function better in work, education, and other everyday situations.

The results of the presented study also indicate that memory impairment in adults with DD only exists in the verbal modality. There were no differences in target or context recollection (either negative or positive) for pictorial material. As compensatory mechanisms emerge in cognition and behavior with individual development, it is possible that in adulthood impairments will be evident only in the domain that is affected by other pathomechanisms (verbal), even if they can also be found in other domains (such as pictorial) at a younger age. Interestingly, the standard analysis shows significant differences in pictorial memory, but multinomial modeling allows us to confirm that these differences are connected not to recollection processes, but response bias. As mentioned earlier, multinomial modeling allows for a more elemental analysis, which in turn can present results patterns that explain unclear or contradictory findings from current or previous studies (*cf*. Obidziński and Nieznański, 2017). The presented study provides another argument for the importance of modeling to better understand DD memory functioning, which should encourage the use of this analytical approach in future studies in the context of various memory theories and processes.

However, despite this lack of difference in pictorial memory, an interesting pattern of verbal and pictorial memory relationships in dyslexia was observed in the correlational analysis. The DD group has stronger correlations between verbal and pictorial memory under similar experimental conditions, and most importantly there is also a significant difference in correlations between the DD and TD groups for measures of memory for stimuli presented in a perceptual context and with the test probe about the perceptual context. This observation corresponds with some theoretical and practical views that there is a greater connection between visual processing and verbal processing in the DD than the TD group (e.g., Bacon and Handley, 2014; Snowling, 2019). This stronger link between verbal and visual processing seems to manifest in the memory parameter correlations observed in the current study. However, the results of our current experiment do not suggest a clear interpretation of this finding, and studies dedicated to this effect are needed. This is primarily due to the fact that the correlation analyses were conducted in separate groups, which lowered their statistical power. If more studies show a similar effect, it may be interesting from a practical point of view to test whether a stronger link between verbal and pictorial memory in dyslexia can be interpreted and used as a compensatory strategy or whether it is more an effect of dyslexia pathomechanisms.

### Study limitations

As stated in the discussion of the results above, it is possible that the specificity of the applied procedure (Nieznański, 2020) leads to unwanted effects when employed in dyslexia research, that is, because of its verbal complexity. This could potentially be the reason for context memory impairment (compared to the TD). Taking into account the fact that this procedure allows not only the verbal and pictorial memory differences but also the differences in perceptual and semantic context processing to be investigated, its use was justified in the case of the dyslexic memory study. Nevertheless, future studies should investigate the verbal context and target recollection using different procedures (e.g., standard DRT procedure: Brainerd et al., 2015) to further investigate context recollection in dyslexia. Another limitation concerns the possibility of assessing correlations between model parameters, which is not available in our method of MPT analysis but is offered by hierarchical MPT analysis (see: TreeBUGS package, Heck et al., 2018). The traditional method of analysis used in the current article is based on data aggregated across participants—so it does not allow for the investigation of such correlations, limiting the ways in which the seventh hypothesis could be tested. Future studies devoted to the relationship between verbal and pictorial memory would benefit from using the hierarchical MPT modeling method.

## Conclusion

On the level of both standard and multinomial modeling analyses, specific patterns of memory functioning for the dyslexic group were observed. Differences were observed for both verbal and pictorial memory and were specific for different types of context and memory processes. Once again (*cf*. Obidziński and Nieznański, 2017), multinomial modeling proved to be a very useful method in dyslexia research, allowing a better understanding of the results of the study’s standard analysis. In dyslexia, both the context and the target recollection of verbal material is impaired; however, significant differences were found only for semantic processing. In the case of perceptual processing, there is no significant difference in this process; however, a difference in bias was observed showing that dyslexic participants are more likely to answer “yes” in the case of pictorial material when they are not sure if the given stimulus was presented. Taking into account the fact that the memory experiment procedure could be more difficult for participants with dyslexia (especially in the verbal memory condition), a future investigation of context and target recollection in dyslexia using less demanding procedures is needed for a better understanding of memory functioning in this developmental disorder.

## Data availability statement

The datasets presented in this study can be found in online repositories. The names of the repository/repositories and accession number(s) can be found at: https://osf.io/wcr5p/?view_only=03570721ea2b49a69bf06f43b92342bd; Dual-recollection of verbal and visual material in developmental dyslexia; doi: 10.17605/OSF.IO/WCR5P.

## Ethics statement

Ethical review and approval was not required for the study on human participants in accordance with the local legislation and institutional requirements. The patients/participants provided their written informed consent to participate in this study.

## Author contributions

MO (80%): conceptual work, recruiting subjects and conducting the study, data processing and analysis, preparation of all parts of the article manuscript, and preparation of the online repository. MN (20%): conceptual work, preparation of the experiment, and work on the introduction and discussion parts of the manuscript. All authors contributed to the article and approved the submitted version.

## Funding

This research was supported by a Grant (no. 2018/29/N/HS6/01833) from the National Science Center, Poland.

## Conflict of interest

The authors declare that the research was conducted in the absence of any commercial or financial relationships that could be construed as a potential conflict of interest.

## Publisher’s note

All claims expressed in this article are solely those of the authors and do not necessarily represent those of their affiliated organizations, or those of the publisher, the editors and the reviewers. Any product that may be evaluated in this article, or claim that may be made by its manufacturer, is not guaranteed or endorsed by the publisher.

## Footnotes

^1^ https://osf.io/wcr5p/
